# Housing and Health in Ghana: The Psychosocial Impacts of Renting a Home

**DOI:** 10.3390/ijerph7020528

**Published:** 2010-02-11

**Authors:** Isaac Luginaah, Godwin Arku, Philip Baiden

**Affiliations:** 1 Department of Geography, The University of Western Ontario, London, ON N6A 5C2, Canada; E-Mail: iluginaa@uwo.ca; 2 Department of Sociology, The University of Western Ontario, London, ON N6A 5C2, Canada; E-Mail: pbaiden@uwo.ca

**Keywords:** housing, advance rent, psychosocial health, coping strategies, Accra Metropolitan Area, Ghana

## Abstract

This paper reports the findings of a qualitative study investigating the impacts of renting a home on the psychosocial health of tenants in the Accra Metropolitan Area (AMA) in Ghana. In-depth interviews (n = 33) were conducted with private renters in Adabraka, Accra. The findings show that private renters in the AMA face serious problems in finding appropriate and affordable rental units, as well as a persistent threat of eviction by homeowners. These challenges tend to predispose renters to psychosocial distress and diminishing ontological security. Findings are relevant to a range of pluralistic policy options that emphasize both formal and informal housing provision, together with the reorganization and decentralization of the Rent Control Board to the district level to facilitate easy access by the citizenry.

## 1. Introduction

The Greater Accra Region, with a population of about 2.9 million, is Ghana’s major economic, administrative, and cultural centre. With an average growth between 5–8% and attracting close to 85% of all investments into commerce, finance, manufacturing, private educational institutions and real estate related activities [1,2], new investments over the past two decades have brought about major changes in urban development, which has expanded in physical size by over 300% [3]. Unfortunately, with a quadrupling of its population in just about three decades, housing production in the metropolis has not kept pace with the phenomenal growth thereby making the city’s already bad housing situation worse both in terms of quality and quantity. About 60% of the city’s residents live in overcrowded, deteriorated, low-income rental accommodations without basic amenities such as sanitation, proper roads, drainage, water and waste disposal systems [4]. Additionally, about 40% of all renting households rent two to three rooms [4], with several households renting single bedroom accommodations. Housing shortages have produced high dwelling densities in the poorest neighbourhoods, with some having as many as 49 persons per dwelling unit and 4.2 persons per room [2]. Given the limited number of amenities such as toilet and bathes, such high dwelling densities tend to put a tremendous pressure on the existing facilities.

The government’s response to the deep seated developmental and housing crisis over the years has involved a series of economic reform policies implemented since 1983 [3–5]. The first batch of reforms (1983–1986) was characterized by stabilization measures designed to reduce short-term imbalances between demand and supply, which normally manifest in the balance of payment and budget deficits. The second batch of reforms (1986–1989) consisted of structural adjustment measures intended to address a wider range of barriers to growth [1]. Generally known as Structural Adjustment Programs (SAPs), the two broad economic measures comprised packages of actions that included reducing inflation, devaluing the currency, downsizing public service, improving finances, privatizing state-owned enterprises, promoting exports, removing subsidies, and reducing government expenditures on social services, including education and health care. The third batch of reforms (1990 onwards) involved full liberalization of the economy and included policy measures designed to attract foreign direct investment, encourage an active role by the private sector, establish export processing zones, and dismantle trade barriers and investment regulations. As part of broader macroeconomic policy changes, the government introduced numerous neo-liberal reforms in the housing sector. Key reform measures centered around the following: (1) withdrawal of the state from direct housing production and financing; (2) stimulating the growth of the real estate sector (*i.e.*, private sector); (3) liberalizing the land markets and building material industry; (4) encouraging the formal private sector to construct rental housing units; and (5) reforming housing institutions. In general, the reforms were intended to open the housing sector to competition, to improve efficiency so as to improve the housing finance system, and to increase housing supply through commercial development, foreign investment, and self-building.

Unfortunately, the policy reform measures along with their associated economic benefits have not been closely mirrored by increases in rental accommodation, hence, the AMA and other major urban centers in Ghana are suffering from severe housing shortages and soaring prices of owner-occupied dwellings. In many ways, rising costs of key building materials, high rates of inflation, depreciating value of the local currency (cedi), and speculative housing activities have acted in concert to produce the soaring prices. For instance, prices of building inputs have increased by over 200,000% since the late 1970s [6]. Consequently, prices of modest houses are unaffordable on a price-to-income ratio. Depending on the geographic location, a two-bedroom house costs approximately $60,000, with three- and four-bedroom houses ranging from $120,000 and $200,000, respectively. Given that Ghana’s per capita income is $400, and daily wage is as low as $2 (1USD = 1.0644 Ghana cedi), such houses are not within the means of the majority of the populace. Studies on housing affordability revealed a price/income ratio of 1:67 for a senior public servant and 1:86 for a university professor [6,7]. With the exception of a few resourceful individuals, homeownership is therefore beyond the reach of most Ghanaians, especially those in urban areas. The vast majority of the populace is subsequently forced to meet their housing need in the rental market, where there is severe supply shortages, hence surging rent.

Adding to rental affordability and availability problems is the nature of the rental payment system which tends to put tremendous pressure on many residents. In contrast to the ‘first and last’ and subsequent month-to-month rent payment system commonly practiced around the world, homeowners in Ghana typically require private renters to pay a lump sum of rent for anywhere from two to five years up front. Such lump sum payments (generally called *advance rent*) can sometimes be the equivalent of two to three years worth of accumulated annual salary. In many instances therefore most renters end up borrowing monies from elsewhere for the substantial down payments. Furthermore, under the pretext of better offers from other people searching for accommodation, homeowners will often ask existing tenants to make additional payments to match or surpass new offers (or to account for inflationary pressures). Failure by the existing tenant to pay this difference could result in eviction with any outstanding balance from previous *advance rent* refunded in lump sum by the homeowner. The combination of rising rental costs, fewer housing opportunities, hyperinflationary pressure and the nature of the rental market system (*i.e.*, *advance rent system*) paints a worrying picture for many renters, and indeed poses potential threats to their psychosocial health and well-being.

Despite the potential public health challenges and the threats to the psychosocial health of residents in the AMA and other urban centres in Ghana, there have been no coherent studies on the psychosocial and perceived health impacts of the rental system. The present study is part of a larger project to investigate the health impacts of housing in the AMA. It reports the findings of an interpretative investigation that was conducted in Adabraka, Accra, between July-September 2008 with two specific objectives:

- to explore the impacts of rental housing market and advance rent system on the psychosocial and well-being of renters; and
- to explore renters coping strategies in relation to their housing needs.

In the rest of the paper, we begin by drawing some insights from the literature on health impacts of housing. This is followed by a description of the study methods. The results, and discussion and conclusions then follow respectively.

## 2. Housing and Psychosocial Health

Much has been written about the impacts of housing on health [8–13]. These studies mostly from Europe and North America, frequently focus on the “direct” and “indirect” effects of housing on health, as well as the “hard” and “soft” effects [14]. This review focuses on the “soft” but direct ways that housing influences health, including tenure status, affordability and availability problems, insecurity, control, and social status perception. Dunn [11] refers to these as the “meaningful” dimension to housing, as opposed to the material aspect.

In regards to health outcomes, the evidence suggests that when compared to renters, homeowners generally have lower rates of mortality, lower long-term illness rates, and better self-perceived and mental health [11,13,15,16]. Others have indicated that homeownership confers psychological benefits by reinforcing homeowners’ sense of security, control, and mastery [17–20]. Beyond homeowners’ degree of control over their accommodations and a secure sense of home, an added benefit is derived from homeowners’ capability to modernize and customize their dwellings, which then enhances their positive perception of home and ontological security. Additionally, the positive effect of homeownership on neighbourhood physical and social environments, which, in turn, may have beneficial health effects has also been documented [21].

In contrast, renting, especially public renting, is associated with a lack of control, insecurity, vulnerability, and higher levels of psychological distress and depression [15,16,22,23]. Renters feel a loss of control because they are restricted in what they can do to their property [9,23], they can be relocated, and as discussed previously, their houses can be repossessed easily [24]. Under such conditions, individuals may have difficulty building relationships, undertaking recreational activities, and paying attention to their children’s well-being. This, in turn, reinforces high levels of stress and stress-related illnesses among renters.

Affordability problems can also be a source of chronic insecurity for lower-income private renters and purchasers facing the possibility of mortgage arrears [11,25,26]. This insecurity impacts general and mental health [11,14]. In most instances, lower-income private renters may be forced to make “trade-offs” to offset the impact of housing costs, such as choosing to live in less expensive areas, renting rather than buying, and making compromises on housing quality and suitability [27]. While the combined disadvantages of renting are important, Robinson and Adams [28] consider housing affordability problems to be the most significant.

Saunders [17] argues that a home functions as a secure base where people can relax, feel free of social constraints, and feel safe from socio-economic unpredictability. Similarly, Dupuis [18] suggests that the home confers “ontological security”, providing a place of constancy in the material and social environment, a place in which the day-to-day routines of human existence are performed, a place where people feel most in control of their lives because they feel free from the surveillance that characterizes life elsewhere, and a place that is a secure base around which their identities are constructed. Furthermore, other authors (e.g., [29]) note that the home is one of the few spaces that owners or tenants can use exclusively.

Frequently, the consequences of uncertainty about housing are fear of having no place to call home and an inability to cope with the health threats posed by housing problems. The combined effects of housing as a stressor on health and well-being can permeate both individual, family and community levels (e.g., [16]). Lazarus and Folkman [30] indicate that an individual response to stress consists of three cognitive appraisal processes: primary appraisal, secondary appraisal, and coping. During primary appraisal, people judge an encounter as irrelevant (when an encounter with the environment results in no harm to personal well-being); benign-positive (when an encounter promotes personal well-being); or stressful. The secondary appraisal process is a coping method assumed in response to a specific primary appraisal. During secondary appraisals, two types of coping strategies may be used: (1) action or problem-focused coping, which represents strategies directed towards management of a problem or removal of the effects of the stressor; and, (2) emotion-focused coping, which represents regulating emotional responses to the problem. Additionally, reappraisal of the stressful situation may occur during the coping process. Reappraisal acts as feedback based on new information or continued influence from environmental stressors [30]. The complex two way transaction between the person and the environment is therefore a process of changing emotions and appraisals. Until recently, coping theories had only been developed for and focused on adults who have experienced extreme life events. However, aided by its flexibility and generalizability Lazarus and Folkman’s theoretical model have been extensively used in many areas including environment and population health studies. The above theoretical discussion is used to understand and explain the findings in this study.

## 3. Methods

Little is currently known about the impact of the rental housing market on renters’ health and well-being and the strategies they adopt to cope with the housing crisis in Ghana. Consequently, we utilized interpretative methodology involving in-depth interviews among tenants in the neighbourhood of Adabraka in the AMA. The present study focuses on the AMA because is it the most urbanized of the three administrative districts within the Greater Accra Metropolitan Area (GAMA) having about 57% of the region’s population. This can be explained by the concentration of majority of the nation’s economic activities, administrative functions, as well as socio-cultural activities within this area. As a result, most renters tend to gravitate towards the AMA because of the difficulty of access to the city center from the peri-urban areas. This tends to put enormous pressure on renting in this area in terms of cost of housing and predisposes renters to maltreatment by landlords. Thus, although GAMA as a whole is currently suffering from severe housing shortages and soaring prices of rental units, the problem is more critical in the AMA than the peri-urban areas of the city.

The aim of the study was to get an understanding of participants’ experiences as they relate to housing, and their associated perceptions of health and well-being within the AMA. To achieve maximum variations in participants’ diverse opinions and experiences, adults tenants (n = 33) of varying ages from 25 to 69 years were recruited through a snowball sampling strategy (See Table 1). This recruitment strategy was adopted because there was no existing systematic database such as tax assessment rolls from which to draw homeowners and tenants. After each interview, participants were asked to suggest people who had lived in the neighbourhood for at least one year, and who they felt could contribute to the objectives of the study. They were then asked to contact and inquire if they were interested in the study. Names of interested participants were passed to the researchers. A potential weakness of snowball sampling is the possibility that it might reflect the biases of those who direct the researcher to other participants or biases may arise because of the limited scope of the social networks to be utilized.

Interviews were conducted by two researchers, one fluent in Ga and Akan (two languages most commonly spoken in the AMA). A checklist of topics (semi-structured and open ended questions) for discussion provided the format for the individual interviews. The checklist went through several stages of review and revisions by the researchers and covered topics including: the nature of the housing struggle; perceptions of *advance rent systems*, health and well-being impacts, and coping strategies. Examples of specific questions include: Could you describe to me your experiences of renting accommodation in Accra? How would you describe your perceptions and experiences with the *advance rent system* commonly practiced in Accra? Could you describe the effects of renting on your health and your everyday life? The checklist was designed to be flexible by allowing new questions to be added during the data collection process. The interviews lasted approximately one hour and were audio-recorded with the permission of the respondents and transcribed verbatim.

The analysis was guided by themes and constructs related to the key research objectives. The key categories of responses for questions asked in the interviews were created prior to line-by-line coding, which is generally considered the most appropriate coding mechanism [31]. This is an interactive and inductive process allowing for the data to direct the development of themes. All interviews were coded using the same coding scheme respectively for tenants and homeowners; this is the most effective way of identifying similarities across and differences between individual responses.

Transcripts were coded into themes of renting experiences, perceptions, and challenges. Key categories were reviewed several times in order to ensure that concepts pertaining to the same phenomena were placed in the same category. Additionally we took a number of specific steps to ensure a consistent analysis of the data [32,33], including the use of a comprehensive topic list. A code-recode procedure was conducted with two weeks separating coding. Furthermore, two investigators independently coded and categorised portions of the transcripts and compared and discussed discrepancies in the categorization process. Taking into consideration that transcripts were translated from Ga and Akan into English, two randomly selected participants were used for member checking as a technique to ensure the validity of the findings and to reduce misrepresentation [33,34].

## 4. Results

The themes that emerged from the data were: housing availability and affordability in the AMA, the *advance rent system,* insecurity and the fear of eviction, the meaning of home, and renters’ coping strategies. Direct quotations from transcripts illustrate selected themes and serve to contextualize the responses. Due to the relatively small sample size, participants names are not provided in order to preserve their confidentially. Only gender (M = Male, F = Female) and age are provided at the end of each quotation.

### 4.1. Residents Experiences of Renting a Home

#### 4.1.1. Housing availability and affordability in the AMA

In the Ghanaian context, the ultimate achievement by a person is to own a home as fulfilment of their cultural obligation to their family and extended relatives. Consequently, a home is associated with status and is a sign of progress in life, especially for men. As a result, many people desire to become homeowners where they can have privacy and feel in control of their lives. This extends to Ghanaians living abroad where they desire to build a home back in Ghana.

Thus, when participants were asked how they feel about renting, nearly all of them presented predominantly negative views. Some described the process as “embarrassing” and “a nightmare” in terms of the time needed to find a rental place and the experiences thereafter. A participant described how he and his family struggled to find their current accommodation:

I came to Accra first from the Western Region… Finding a place for my family was almost impossible. My wife and I have four children, so we needed at least a two bedroom rental space… In the end I have to pay my landlord an advance rent of ¢5.6 million which he used to complete the rooms we are now living in…

(M, 41)

As indicated in the comment above, the difficulty in finding a place to live has resulted in people paying *advance rent* for uncompleted buildings. The homeowners then use such monies to complete their properties for waiting tenants, yet this does not guarantee that initial contractual agreements will be adhered to by the homeowner.

Many residents rightly attributed the rental shortages to the rapid population growth in the city, as “more and more people are moving into Accra”. Migration to Accra stems from the biased policies toward the region where majority of the country’s institutions, economic activities, and infrastructural facilities have traditionally been concentrated. As a result, people migrate to the region in search of employment opportunities, schools and health services, and public facilities and amenities creating a “brain drain” in the less-favoured regions.

Other participants explained that housing shortages may have been due to the “new norm of converting residential units into office space and shops”. This new practice is in response to growth in commercial and retail activities over the last few years and the fact that “shop owners are usually less likely to default in rent”.

#### 4.1.2. The advance rent system: a silent killer

By far, participants talked extensively about the *advance rent system* currently practised in almost all Ghanaian urban centres. A few participants had positive perception about the system which they believed “provides a considerable level of stability and long term shelter in [their] life” because renters “do not have to struggle to pay rent on month-to-month basis.” However, the majority admitted that meeting *advance rent* obligations was the major problem in their everyday lives. As one of the respondents stated:

I don’t want to think about my next advance rent because it is quite soul destroying … It is a stressful thought and it always gives me headache... It makes me really sick, and I have no idea what I will do…

(F, 43)

Other participants made comments along the same lines that: “I cannot sleep in the night because I’m always thinking of how to raise money for my next advance rent.” Several participants talked at length about the persistent worries and lack of sleep because of *advance rent*. One male respondent talked about the amount of time he spent working to save enough for his next *advance rent*:

My next rent advance is due in January … Fulfilling this responsibility is my greatest worry now. So I’m working all day and all night to raise sufficient money for my landlord.

(M, 49)

Some participants who were mid-way in their *advance rent* arrangement were already beginning to worry about the expiration of their current rental contract. This fear and stress results from the uncertainty they face for raising another two to three-year *advance rent*. For example, one participant noted the following:

I have two years left in my current contract and the landlord has not told me anything … I am beginning to be distressed about this. We have three kids and all our income goes to cater for their needs, including school fees. We have no savings, and I and my wife are already worrying where we can get up to ¢8 million for advance payment. Even if the landlord will let us stay, we still need this lump sum. We are getting distressed where we are going to get this money.

(M, 51)

For some participants, the expiration of their rent contractual agreement is a constant reminder of the gravity of the problem ahead of them. For those who have not yet finished paying the initial monies they borrowed for their present accommodation, thoughts of raising new *advance rent* cannot be described in any small terms. Another participant who borrowed her initial *advance rent* money and has yet to finish paying described how this might have resulted in her becoming hypertensive:

The first loan I took to rent this place is still outstanding, and I have less than two years remaining. Already I’m beginning to worry about where I’ll get money to renew my contract. I know my landlord will ask for an advance rent again, since month-to-month renting system seems to be a thing of the past in Accra…I discovered two months ago that I am now hypertensive. I never had this problem before, but since coming to Accra, things have been extremely difficult. It is killing me silently…

(F, 43)

The inability to pay *advance* rent often results in eviction. Consequently, most participants associated the *advance rent system* with a high level of insecurity and fear.

#### 4.1.3. Insecurity and the fear of eviction

Many interviewees talked about feeling insecure in their present rental space “because they don’t know when their homeowner will ‘jack up’ [raise] rent” or issue an “eviction order” when they are unable to meet their new *advance rent* obligations. The constant anxiety about been evicted contributes to more stress and ill-health:

I don’t have peace of mind because I’m always worried about being asked to leave. In fact, the risk of losing my chamber and hall [self-contained unit] is a constant concern. I feel so insecure because anything can happen. The landlord has already asked one tenant and his wife to leave… I’ve no control over this … It is very depressing, but what can I do…

(M, 30)

Other participants noted that insecurity is a major obstacle to long-term life planning. A younger participant commented that:

I’ve been thrown out two times over the past three years because I couldn’t pay the additional advance rent my landlords requested. Really I cannot plan anything in life because I don’t know when else I’ll be thrown out of here. I cannot accumulate any assets. In fact, it is useless planning anything when you don’t know where you are going to live a few months from now.

(M, 26)

Another participant described how a single eviction impacted her family’s life.

Our landlord evicted us because we couldn’t afford a two-year *advance rent*. I moved to live with my sister but my husband didn’t find anybody to live with so he moved into our kiosk [wooden shop]. We took our three children to their grandmother in the village. We both plan to work hard, save money and rent a different place together. But the whole mess affected our marriage too…

(F,51)

Throughout the interviews, participants narrated negative and compelling stories to demonstrate their sense of helplessness and their everyday life struggles as they attempted to meet their shelter needs. In effect, most of the participants seem to have lost the meaning of the spaces in which they live.

#### 4.1.4. Renters meaning of home in Accra: A loss of ontological security

Respondents in the study talked at length about what their various housing arrangements mean to them as places they should be calling ‘home’. In fact, there was no single participant who described their ‘home’ in reassuring terms like site of constancy, security, privacy, and mastery [11,17,18]. Instead, the difficulty of meeting rental commitments and the persistent fears of “being asked to move out” seems to have overwhelmed many renters in the AMA and left them describing their ‘homes’ as meaningless places in their everyday lives. As one participant stated, “Home for me is like a living in hell and I have no emotional attachment there”. Similarly, others agreed that their homes do not confer privacy because of congestion and the frequent surveillance from their homeowners. Some participants talked about this passionately:

I don’t have any privacy at home because there are too many people sharing the spaces … toilet, bathroom, kitchen … How can one have privacy when you have to share these places with so many people?

(F, 40)

I don’t think I have control of my life in this home. I cannot do what I want and when I want it because there are too many people around at all the time … I wish I do though.

(M, 33)

Privacy at home? No, no, no, my landlord is always spying on me … to see how many people are in my room … who is visiting me … even what I’m cooking or eating. I feel like I’m under constant surveillance, and I’m not able to live a normal life.

(M, 27)

It was disturbing to hear some participants indicate that they had “more peace at work” than at their homes, and the consideration that home should serve as a retreat from daily socioeconomic unpredictability was nonexistent among respondents [35]. Overall most of the participants agreed that impact of rental obligations such as raising the lump sum payment (*i.e.*, *advance rent*) in particular, not only lead to deteriorating psychosocial health problems, but is also considered as a “silent killer” in the general population. Nevertheless, residents try various ways to manage or cope with their situation.

### 4.2. Coping Strategies

The discussion about coping with the housing situation in Accra centred around the difficulties in accumulating and paying advance *rent*. Residents invoke both action and emotion focused coping strategies [30]. Action focused strategies involved problem solving behaviours (such as support from family and friends; working hard to save; co-renting; dialogue), and emotion-focused strategies (accepting responsibility; denial; escape avoidance; confrontive; reliance on God) in order to cope with their housing needs in the AMA.

#### 4.2.1. Action focused strategies

Several participants talked about how they try to cope with the housing situation by mobilizing family resources and making use of their social networks. These include “seeking financial assistance from immediate and extended relatives” and “borrowing money from friends”, especially those living abroad. As one participant explained:

Through personal savings I was able to raise half of the money … this was not enough… but by the grace of God my brother who lives abroad in the United States assisted me with the rest of the money.

(F, 44)

Study participants acknowledged that *advance rent* commitments create a major life struggle and frequently result in severe financial constraints for a range of household expenditures, including food, children’s school fees, and many basic necessities as they “try to save money for future advance rent”:

I was able to raise sufficient *advance rent* by working very hard … I work all day at the store … so also is my husband, we had no choice but to do that in order to raise enough money for our advance rent…now we are focused on saving some money as we have only a year left before the landlord will start asking for a new rent contract…

(F, 48)

Other participants indicated that they try to cope by making conscious “trade-offs”, such as “sacrificing housing space for a lower cost” and “spending very little or nothing on non-essential items”. Participants explained that such trade-offs were a priority even though they recognize the negative effects on their quality of life. As one woman explained:

[My husband and I] acknowledged our situation and we try to put things into proper perspective. We know we don’t have the money so we try to live very simple lifestyles. We’ve decided not to spend by-heart [avoid unnecessary spending]. We avoided spending on expensive clothing, … no unnecessary shopping. We have also limited the use of our cell phones and stuff like that … Our main objective is to be able to meet our advance rent obligation next year.

(F, 48)

Apart from working hard to save money for housing or implementing trade-offs in order to cope, some participants indicated they try to dialogue with their landlords to rearrange rent payments. Others indicated they co-rent (two or three people combining to rent an accommodation) with others. Most residents agreed that they hope to have their own homes one day. An interviewee commented that:

I have been working very hard and my wife too… All we have been hoping to do is save some money and buy a plot so that we start building slowly, slowly… I know that with God, one day, we will be living in our own house …

(M, 50)

The study participants also revealed that it was getting more and more common to find people co-renting the same living space, often relatives or friends. This strategy is primarily practiced by single adults in their twenties and thirties. For instance, a male participant described his experiences:

… well, I couldn’t raise rent advance all by myself. At the same time two of my friends were facing severe financial hardships and could not raise their rent advance. I suggested to them that rather than living separately we should combine our resources and stay together. They both liked the idea so we are now sharing ... Sure, if any of us gets married, then you find your own place ... We’ve been able to reduce our expenses substantially because we share everything together, including utility bills, food ... we do not have absolute privacy, but hey, what can we do?

(M, 25)

Beyond action-focused coping strategies, participants also used emotion-focused strategies to deal with the impacts of housing issues in Accra.

#### 4.2.2. Emotion focused strategies

Some of the people in the AMA are coping with the housing situation by accepting it and therefore resigning themselves to “just having to endure what is happening”. One of our female respondents explained that if you live in Accra these days, you have to understand that tenancy arrangements are always temporary at best:

It’s a real struggle but you just have to live with it … The housing struggle is getting tougher with each passing day. Unfortunately, the government is not doing anything about it. It’s tough but we’ve got to cope with it … one has to accept whatever it is and live your life…

(F, 51)

Similarly, another interviewee talked about the need to cope since housing problems can beset anyone without their own home in the AMA:

Whatever will happen, I have no control over it, I just have to accept it and move on … In life you always have to expect the worse… I know friends who have been kicked out of their accommodations for no reason all simply because they did not have extra money to add to the advance rent they already paid the landlord … Do I think that can happen to me too? Yes, and if it happens, I don’t have my own home … so I have no other choice than to try and deal with it …

(M, 38)

While others supported the preceding views on accepting responsibility, the gravity of the situation has led some respondents into denial.

Some residents ‘turn inwards’ [36,37] by denying the associated impacts of the housing crisis on their health as well as the well-being of their families. For instance, while many residents admit that the rental situation in Accra is a nightmare, others reported that they frequently ignore the threats from their landlords since they don’t believe they can be thrown out of their accommodation without committing any offence:

I just moved into my present accommodation… less than a year ago and I paid ¢5.2 million advance rent for three years. I am hoping the landlord will not come anytime soon to ask me to move. Besides, we are four tenants and if someone will have to move, it can’t be me… I don’t see why he should be thinking about kicking me out…

(F, 46)

The complexity and stressful nature of the situation means that some participants are coping spiritually by focusing on the power of God, and His divine intervention in their current housing situation:

The situation in Accra is something only God knows … It is a bad situation and if you think about it too much, you will probably die of too much stress … It is very depressing. All I do is pray everyday that God can protect me and my family and also touch the heart of my landlord so that he does not come asking for more money or saying that we should move, and I know God is in control of our situation …

(M, 53)

In the current situation it not surprising that some tenants in the AMA have desperately resorted to ‘escape avoidance’ in order to cope with their housing problems.

Other participants cope by avoiding or hiding from the landlord. Typically such tactics starts with some form of attempt to negotiate with the landlord to be in default for one or two months, or to pay only a portion of whatever they owe in terms of rent. In situation of unsuccessful negotiation, the participants then resort to hiding from their landlord. A participant explained that “when you do not have the money for the landlord or landlady, you have no choice but to try to avoid him or her while looking at other options”. A male participant recounted what he does:

I often woke up early [about 4 a.m.] to go to work and came home very late when they [landlord and family] are sleeping. As much as possible I tried to avoid seeing the landlord in the house or anywhere in the neighbourhood. In fact, I never saw the landlord for about a month. I know this cannot go on forever, and I’m very worried about it … I’m doing my best to find the money … I’m hoping I will not come home one day and see my things [belongings] outside the door.

(M, 35)

Those who resort to avoidance as a coping strategy run the risk of being “kicked out” of their rental space, a risk that frequently results in confrontational measures including: “picking up quarrels with homeowners”, “ending up in court”, and “making official reports to the Rent Control Board (RCB)”—a state agency mandated to resolve disputes between homeowners and tenants. However, it was clear from the interviews that the participants were disgruntled with the operations of the RCB because of the length of time it takes to resolve cases, perceived corruption associated with their activities, and a lack of executive power to enforce rules and regulations [38].

## 5. Discussions and Conclusions

The findings here clearly show that renters in the AMA operate within conditions of considerable constraints including the difficulties of finding appropriate rental units at reasonable prices. The housing sector continues to suffer persistent decline over the past years, even at a time when Accra and other major cities across the country are experiencing population influx from the rural areas. In part, the decline is due to the continuing state’s withdrawal from direct provision of housing [39], and the current form of housing production by private investors which focus primarily on home-ownership. Consequently, the bulk of rental accommodation is provided by the “household sector”, which goes almost unrecognized in policy terms. Thus the combination of fewer housing opportunities, skyrocketing rental costs, and lump sum rent payment makes the rental market a very challenging environment for renters in the AMA. As a result of the mismatch between the supply and demand of rental accommodation, homeowners have taken advantage and have adopted the *advance rental system*.

The latent effects of the trade-offs that residents have to make to raise sufficient money to pay *advance rent* in order to avoid being evicted is of major concern. Such trade-offs includes sacrificing housing space for lower cost, spending very little on children and their education, lack of quality food, and lack of family activities that provide exercise and emotional stability, may adversely affect residents’ quality of life and result in poor health status [11]. Furthermore, parents working extended hours or staying away from ‘home’ may have difficulty developing a sense of attachment with the home environment. This is unfortunate since an attachment with one’s home environment has been associated with better health status [40].

The development whereby co-renting accommodation is on the increase in the AMA and other Ghanaian urban centers is worth noting, since co-renting compromises individuals’ privacy, and may negatively impact residents’ mental health [35]. Co-renting may also result in overcrowding which could facilitate transmission of infectious diseases.

In light of these challenges, there is a growing lack of ontological security [18], with many renters attaching very little meaning to the places they should be calling ’home’. Saunders [17] indicated that ‘home’ is a place where people can relax, feel free of social constraints, and feel safe from socio-economic unpredictability; however the findings demonstrate that these appear to be nonexistent among renters in the AMA. In fact, there was no single renter in the study sample who described their ‘home’ in reassuring terms like site of constancy, security, privacy, and mastery [11,16–18].

The findings point to widespread psychosocial distress as a result of the daily housing struggle. This underscores Mueller and Tighe’s [26] argument that the difficulty of meeting housing obligations, particularly by low-income groups, can primarily be a source of chronic stress, and may often lead to additional secondary stressors on renters and their families (see also [28,30,41]). Indeed, the anxiety and stress associated with renting a ‘home’ has made paying adequate attention to children and other daily necessities difficult. Renting a home in the AMA seems to be subjecting renters to intense psychological distress, with no positive health benefits [35].

Although it was clear that renters in Accra pursue a number of coping mechanisms to address their housing challenges, such measures seems to be falling short of relieving them from poor psychological health, and may be manifesting into other more chronic health problems with damaging consequences. For instance, due to the amount of time spent working to save for *advance rent*, some parents not only had difficulty developing social relations, but more so paying sufficient attention to their children’s welfare, most notably in the area of education. This is consistent with Leslie’s [42] assertion that the anxiety and stress associated with affordable housing can contribute to child neglect, which leads to children becoming difficult for parents to handle.

Appropriate housing has long been identified as an important health resource and one of the basic necessities of life; however the issue has not attracted adequate academic and policy attention in Ghana. Indeed, more often housing has been treated as an individualized issue, and an indicator of wealth rather than a health promoting resource. In this regard, this study provides good quality information on families’ housing struggles and the impacts on their psychosocial health and well-being. Considering the strong socio-economic similarities between Accra and other rapidly expanding cities in the developing world, the findings may be generalized to some of these areas.

Overall, findings revealed that the difficulty of renting a home in the AMA is a potential public health issue that needs clear and practical policy attention, unfortunately to date, housing problems seem to be eclipsed by misplaced policy priorities and persistent corruption. As with most policy issues, political ‘lip service’ abounds on providing housing for the poor and low-income Ghanaians, but very clear actions are rare to find. We suggest a range of policy options on the critical connections between housing and the psychosocial health of Ghanaians. As a starting point, national policy needs to recognize the interrelationships between housing and health, in particular, understanding that housing is a key determinant of health [11,16]. This could then lead to comprehensive policies aimed at encouraging a range of housing forms, tenures and delivery agents that contrast the unacceptable national housing policy that focuses primarily on home-ownership. Specifically, the Ghanaian government must shift its policy focus from an emphasis on formal sector providers to include others such as private, community, NGOs, and most importantly, the individual builder sector [43].

This recommendation is in line with Keivani and Werna’s [44] call for a pluralistic approach in housing the poor in developing countries. Within this approach, the state should serve as an enabler, while acknowledging contributory roles of other providers. There is also a need for policy to recognize the operation of the household rental sector and to incorporate the sector into a broader housing policy. In line with this, a reorganization of RCB, including decentralizing its functions to the district level is warranted. This will make the RCB more accessible to the general public. Based on the renters’ comments and experiences, we support Yeboah’s [43] argument that any policy approach to resolve the housing crisis in Ghana that does not tackle land issues aimed at making land accessible to the poor one way or the other, is bound to fail.

On a broader scale, the current inter-regional inequality calls for a re-thinking of traditional approaches to physical infrastructure investment, economic development, social policy, and regional planning. Within this context, the recently announced Savannah Accelerated Development Program which aims at reducing poverty in the three northern regions of the country is laudable. Such broad-based regional policy is required for other less developed regions to help address interregional socioeconomic disparities within the country. In conclusion, the household sector in the AMA is undoubtedly playing a crucial role in accommodating a significant element of the populace. The challenges plaguing the sector can be addressed, but this requires recognition by policy makers that housing is far more complex than simply a physical shelter; it is an important health resource with related issues of affordability and accessibility having significant potentials to impact the psychosocial health and well-being of individuals and families.

## Figures and Tables

**Table 1.** Characteristics of the study sample.

Characteristics	Tenants
Sample size	33
Gender	
Male	18
Female	15
Marital status	
Married/ living with partner	17
Widowed/Separated/Never Married	16
Mean age	41.9
Education	
No Formal Education/Non-Formal Education	3
Completed Primary Education	4
Completed Middle/Junior Secondary School	14
Completed Senior/Secondary School	9
Completed College/Post-Secondary/Bachelors	3
Average Number of Years in Community	4
